# Resveratrol-Encapsulated Mitochondria-Targeting Liposome Enhances Mitochondrial Respiratory Capacity in Myocardial Cells

**DOI:** 10.3390/ijms23010112

**Published:** 2021-12-22

**Authors:** Takao Tsujioka, Daisuke Sasaki, Atsuhito Takeda, Hideyoshi Harashima, Yuma Yamada

**Affiliations:** 1 Department of Pediatrics, Graduate School of Medicine, Hokkaido University, Kita-15, Nishi 7, Kita-ku, Sapporo 060-8638, Japan; tjoktko@med.hokudai.ac.jp (T.T.); d.sasaki0304@med.hokudai.ac.jp (D.S.); a-takeda@med.hokudai.ac.jp (A.T.); 2 Faculty of Pharmaceutical Sciences, Hokkaido University, Kita-12, Nishi-6, Kita-ku, Sapporo 060-0812, Japan; harasima@pharm.hokudai.ac.jp

**Keywords:** mitochondria, myocardial cells, liposome, mitochondrial delivery, mitochondrial respiratory capacity, resveratrol

## Abstract

The development of drug delivery systems for use in the treatment of cardiovascular diseases is an area of great interest. We report herein on an evaluation of the therapeutic potential of a myocardial mitochondria-targeting liposome, a multifunctional envelope-type nano device for targeting pancreatic β cells (β-MEND) that was previously developed in our laboratory. Resveratrol (RES), a natural polyphenol compound that has a cardioprotective effect, was encapsulated in the β-MEND (β-MEND (RES)), and its efficacy was evaluated using rat myocardioblasts (H9c2 cells). The β-MEND (RES) was readily taken up by H9c2 cells, as verified by fluorescence-activated cell sorter data, and was observed to be colocalized with intracellular mitochondria by confocal laser scanning microscopy. Myocardial mitochondrial function was evaluated by a Seahorse XF Analyzer and the results showed that the β-MEND (RES) significantly activated cellular maximal respiratory capacity. In addition, the β-MEND (RES) showed no cellular toxicity for H9c2 cells as evidenced by Premix WST-1 assays. This is the first report of the use of a myocardial mitochondria-targeting liposome encapsulating RES for activating mitochondrial function, which was clearly confirmed based on analyses using a Seahorse XF Analyzer.

## 1. Introduction

Nanoparticles, a type of drug delivery system (DDS), have some advantageous and unique characteristics, including enhanced biocompatibility and availability, reduced side effects of the encapsulated drugs and the delivery of a drug to specific cells or tissues [1]. Although stem cells, exosomes, growth factors and siRNA are emerging as potential therapeutic candidates, these by themselves face some difficulties and have failed to proceed to clinical use [2,3]. This underscores the new and safer ways to deliver therapeutic agents in order to enhance their bioavailability. Nanoparticles have the potential to meet these needs. This nanotechnology can also be used as diagnostic and therapeutic methods for the treatment of cardiovascular diseases, examples of which include the repair of a post-infarct myocardium and protection of ischemia-reperfusion injury [1,2,3].

Evidence that nanoparticles can be used for cardiac repair and cardioprotection has recently appeared, and liposomes (LPs) have emerged as promising candidates for the delivery of therapeutics into the infarcted heart, and have been already shown to be efficient in preclinical studies. It has also been demonstrated that an LP treatment reduced tissue infarct size, fibrosis, heart failure and mortality in animal models [1,2,3]. In addition, we now have a better understanding of the pathophysiology of reperfusion injury of the ischemic heart, in which the opening of the mitochondrial permeability transition pore is recognized as one of the major contributing factors [4]. Indeed, mitochondria-targeting LPs were reported as having the potential for being efficacious in studies using myocardial ischemia-reperfusion injury models [5,6].

In our previous studies, we reported on the development of our original mitochondria-targeting LP, MITO-Porter [7,8], and on its use in delivering various cargoes to mitochondria as well as the therapeutic efficacies of this DDS [8,9,10]. However, our MITO-Porter had a low cellular uptake activity by H9c2 cells (cardio myoblast cell line). We also developed another LP, “multifunctional envelope-type nano device for pancreatic β cells (β-MEND)”, which was originally designed to target pancreatic β cells [11]. We recently reported that this β-MEND had a higher uptake efficiency for myocardial mitochondria than the original MITO-Porter [12], and that this system appears to have the potential for use in myocardial cell therapies.

Resveratrol (RES), a naturally occurring polyphenolic compound belonging to the stilbene family, has been shown to have beneficial effects in human health, including anti-cancer, cardioprotective, antioxidant and anti-inflammatory activities [13]. The cardioprotective effect of RES is not only due to its antioxidant and anti-inflammatory properties, but also by its ability to modulate enzymes, cell signaling pathways and gene expression [14], which are associated with cellular apoptosis and metabolism, structural or electrical remodeling and the transport of Ca^2+^ [15]. RES has also been reported to show efficacy in myocardial injury models by targeting mitochondria [6,16,17,18]. For example, this efficacy was mediated by eliminating mitochondrial ROS, decreasing mitochondrial membranous permeability transition pore opening and blocking mitochondria-dependent apoptotic pathways in a hypoxia-reoxygenation injury model [6], or by upregulating the activity of sirtuin 1 and AMP-activated kinase [17].

**Figure 1:**
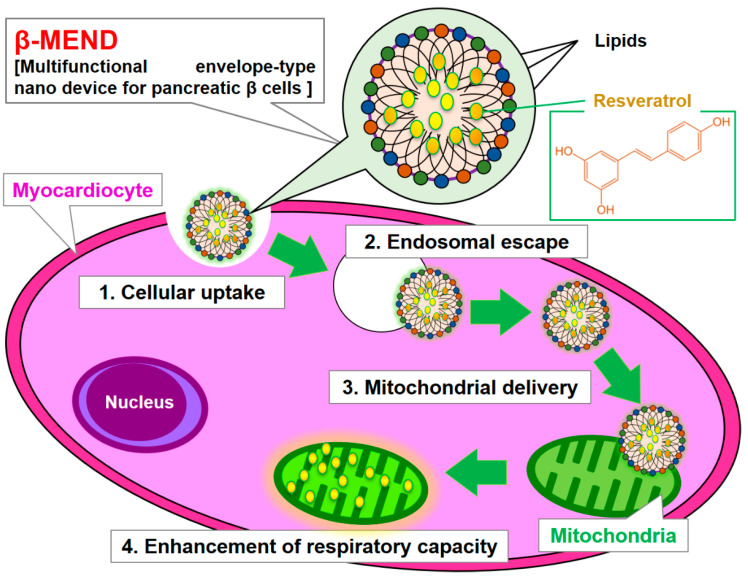
Schematic diagram of the mitochondrial delivery of RES. RES is encapsulated in cationic and mitochondria-fusogenic liposomes. Multiple β-MENDs are taken up by cardiomyocytes, and some escape into the cytoplasm where the positively charged β-MEND binds to negatively charged mitochondria via electrostatic interactions. The delivery of RES to the myocardial mitochondria results in an enhanced mitochondrial respiratory capacity.

In this study, we report on an investigation whether our β-MEND, which contains encapsulated RES (β-MEND (RES)), enhances myocardial mitochondrial respiratory capacity and, if so, to what degree. It was expected that the β-MEND would be internalized by cardiomyocytes (H9c2 cells) and then reach the myocardial mitochondria via electrostatic interactions between the positively charged β-MEND and negatively charged mitochondria. The β-MEND (RES) was found to reach myocardial mitochondria, resulting in an increase in mitochondrial respiration (Figure 1).

## 2. Results

### 2.1. Property of LPs

The β-MEND (RES) was prepared by the lipid film hydration method, and an empty β-MEND was also prepared at the same time as a control. Their properties were evaluated by means of a Zetasizer Nano ZS.

As shown in Table 1, the mean diameter, the polydispersity index (PDI) and ζ-potential of β-MEND (RES) were 79.7 ± 10.2 nm, 0.26 ± 0.03 and 37.7 ± 8.6 mV, respectively. The positive charge of the β-MEND is due to the cationic characteristics of 3β-[N-(N′,N′-dimethylaminoethane)-carbamoyl] cholesterol (DC-Chol), as previously reported [11]. The properties of the empty β-MEND were comparable to that of the β-MEND (RES).

### 2.2. Evaluations of LP Uptake by Fluorescence-Activated Cell Sorting (FACS) Analyses

The uptake of the LPs into H9c2 cells was evaluated by FACS analyses. After a 1 h treatment, the H9c2 cells were collected and analyzed by CytoFLEX. The cellular uptake of the β-MEND (RES) and the empty β-MEND are expressed as the mean fluorescent intensity (MFI).

**Figure 2:**
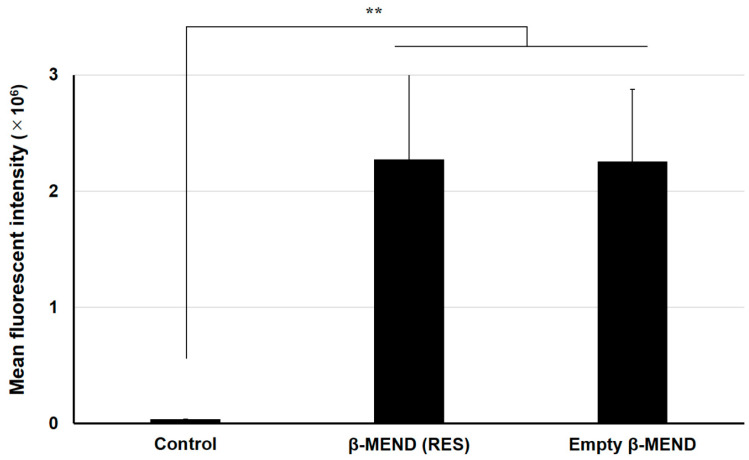
Fluorescence-activated cell sorting analyses of the cellular uptake of β-MENDs. The LPs were labeled with DiI, a fluorescent dye, and the cells were analyzed after the liposomal treatment. Cellular uptake is expressed as the mean fluorescent intensity, an integral value of the fluorescent intensity and cell counts. Data are represented as the mean ± SD (n = 3). Significant differences were calculated by non-repeated measures ANOVA followed by SNK test (** *p* < 0.01).

The MFIs of the β-MEND (RES) were significantly elevated compared to non-treated cells (control). That of the empty β-MEND was also significantly increased more than the control, which was not significantly different from the β-MEND (RES) (Figure 2). These results indicate that the uptake of the β-MEND by the H9c2 cells is not influenced by the presence of RES.

### 2.3. Evaluation of LP Uptake by Confocal Laser Scanning Microscopic (CLSM) Observations

The intracellular localization of the β-MEND (RES) and the empty β-MEND was observed by CLSM. For the CLSM observations, the H9c2 cells were incubated with the LPs for 1 h. After 2 h, the mitochondria were stained with Mito Tracker Deep Red (MTDR), and the media was then replaced with media that did not contain phenol red.

**Figure 3:**
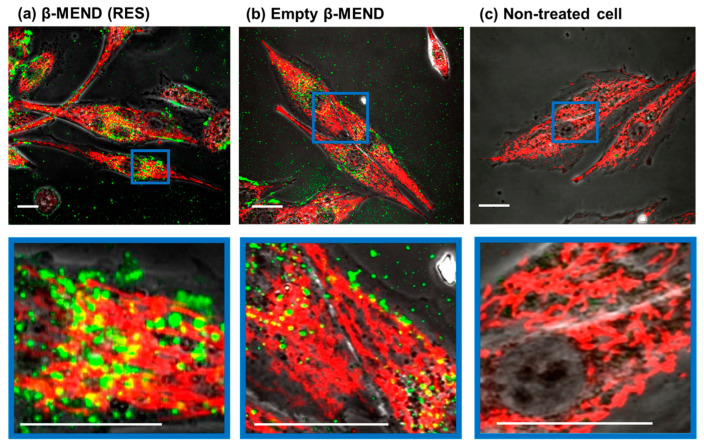
CLSM observations of the cellular uptake of the β-MENDs. The LPs were labeled with an NBD-DOPE, a fluorescent green dye. The cells were observed by CLSM after being incubated with the β-MEND (RES) (**a**) and the empty β-MEND (**b**), and the mitochondria were stained with Mito Tracker Deep Red. The non-treated cells were also observed (**c**). The colocalization of β-MENDs with mitochondria are indicated as yellow clusters. The panels below show higher magnification images framed by blue lines. Scale bars = 20 μm.

The CLSM observations revealed that the green-stained β-MEND (RES) was taken up by the H9c2 cells at the same level as the empty β-MEND. Some of the intracellular LPs were also colocalized with red-stained intracellular mitochondria, which were colored yellow (Figure 3). These findings were considered to be invariable regardless of RES-loading or not.

### 2.4. Measurement of Mitochondrial Respiratory Function

After the β-MEND (RES) had been internalized and reached the myocardial mitochondria, we assumed that the delivered RES would then have an influence on mitochondrial respiratory function. The mitochondrial respiratory capacities calculated as the oxygen consumption rate (OCR), based on Seahorse XF Analyzer assays, were compared between non-treated controls and treated with naked RES and the β-MEND (RES). The concentrations of the drugs in each well were all fixed to being equivalent to 10 μM of RES.

**Figure 4:**
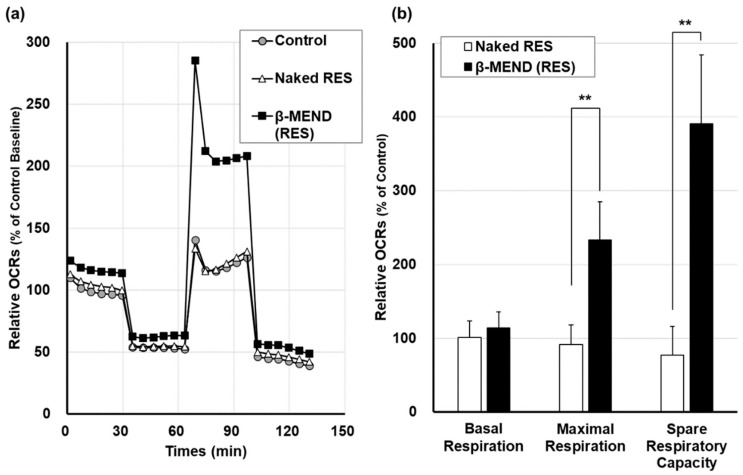
Evaluation of mitochondrial respiratory capacities on the β-MEND treatment. Mitochondrial respiratory activities were evaluated by means of a Seahorse XFp Analyzer after a 3 h treatment with the β-MEND (RES) and naked RES. (**a**) The relative oxygen consumption rates (OCR) ratios were calculated by normalizing by cell counts and mean OCRs at the baseline of non-treated control group. Each mark represents the mean (n = 3). (**b**) The parameters of the relative OCR ratios were calculated by normalizing the counts for each cell and the mean OCRs of the control. Data are the mean ± SD (n = 3). Significant differences were calculated by non-repeated measures ANOVA followed by SNK test (** *p* < 0.01).

When all OCRs were expressed as evidenced by a plotted line graph, the peak of the β-MEND (RES) group appeared to be elevated compared to the others (Figure 4a). The basal respiration in the β-MEND (RES) group was 114.1 ± 21.0% of the control, which did not represent a significant increase when compared to non-treated controls and the naked RES group. While the maximal respiration and the spare respiratory capacity in the β-MEND (RES) group were significantly elevated compared to the control group and the naked RES group, both of these parameters were essentially unchanged in the naked RES group (Figure 4b). These findings indicate that the β-MEND (RES) affected the peak of the mitochondrial respiratory capacity while baseline capacity was not influenced.

### 2.5. Cytotoxicity of the LPs

**Figure 5:**
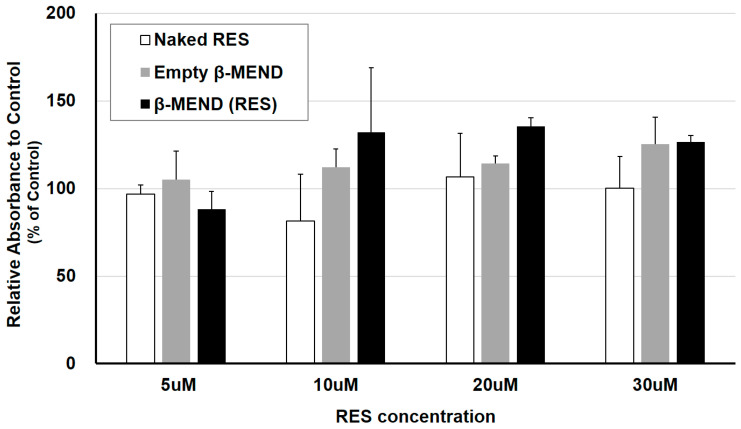
Evaluation of the cellular toxicity of β-MENDs. Cellular viability was analyzed as the absorbance after liposomal treatment using the Premix WST-1 reagent. Each concentration described means the final concentration of RES in the naked RES group, the final equivalent concentration to RES in the β-MEND (RES) group and the same concentration of lipid as in the β-MEND (RES) in the empty β-MEND group. The relative absorbance was calculated by normalizing by that of the control. Data are the mean ± SD (n = 3).

The cytotoxicity of the β-MEND was evaluated by a Premix WST-1 assay. In viable cells, this reagent is reduced by an enzyme in the mitochondrial respiratory chain, and the cellular viability is measured as the absorbance of the reduced reagent. The absorbance of the H9c2 cells that had been treated with the naked RES and the empty β-MEND, and the β-MEND (RES) was compared to that for the non-treated control. As shown in Figure 5, cellular viability for both the empty β-MEND group and the β-MEND (RES) group did not decrease. This demonstrates that the β-MEND is not toxic (i.e., has no effect) on the viability of H9c2 cells.

## 3. Discussion

The findings reported in this study confirm that the β-MEND (RES) was efficiently taken up by H9c2 cells and showed no toxicity; this resulted in enhanced mitochondrial respiration, as evidenced by a Seahorse XF analysis. This suggests that RES has the ability to activate mitochondrial respiratory capacity in cardiomyocytes.

Our previous study reported that a MITO-Porter demonstrated a poor uptake by myocardiocytes [12]. Because of this, we delivered RES via the MITO-Porter to cardiac progenitor cells as models of a doxorubicin-induced myocardial injury, and we observed a promising therapeutic effect [18].

Several investigators previously reported on a therapeutic effect by RES for doxorubicin-induced myocardial injuries [19,20,21,22]. Cheung et al. examined the mechanism responsible for the delivery of RES, which demethylated ATP synthase in mitochondria and the electron transport chain complex or upregulated cardiolipin in the inner membrane of mitochondria by activating sirtuin 3 in the mitochondrial matrix, resulting in an enhanced mitochondrial respiratory function [23].

Based on the Seahorse XFp Analyzer assays used in this study, our β-MEND (RES) caused a significant increase in maximal respiration and spare respiratory capacity in H9c2 cells, while basal respiration was not increased. Although the majority of studies on the respiratory function of myocardial mitochondria using this assay reported that the changes in basal respiration were correlated with those of maximal respiration and spare respiratory capacity [23,24,25,26,27,28,29], others have also reported the same changes on OCRs as we report here. Cheung and colleagues reported that the introduction of the short-form of the sirtuin 3 gene into cardiomyocytes resulted in an elevation in maximal respiration and spare respiratory capacity without a significant increase in basal respiration [23]. A study by Wanka et al. reported that cyto-renin, one form of proteins from the renin transcripts, enhanced the maximal respiration and the spare respiratory capacity of mitochondria in H9c2 cells with no change in basal respiration [30]. Since the mechanism responsible for this is not known, this represents an important issue that needs to be further investigated.

The question arises as to what is the significance in the elevation of the only peak of OCRs evaluated by the Seahorse XFp Analyzer without a change in baseline values. It is known that spare respiratory capacity is decreased in the heart under conditions of severe stress, such as an increased load by a catecholamine infusion, oxidative stress or ischemia, resulting in cell death [31,32]. Additionally, a spare respiratory capacity indicates the capacity of oxygen consumption available to cardiomyocytes under conditions of an increase in the demand for ATP or stress, and in which tissue function and cell repair are maintained. Accordingly, it is possible that the increase in spare respiratory capacity plays a protective role for myocardial cells under pathological conditions such as elevated oxidative stress [33].

There are several limitations to this present study. First, in the FACS analyses, Seahorse XF analyzer assays, Premix WST-1 assays, only a small number of samples were examined and this might affect our findings. Second, we were only able to take CLSM pictures with a low visibility for the colocalization of β-MENDs and mitochondria. We hope to continue to acquire higher resolution images. Finally, H9c2 cells were used as models in this study. These cells are immature myocardioblasts and have fundamentally different characteristics from mature myocardiocyte, which we would actually be treating in real live situations. We chose this immature model because of its ease in culturing as a first trial. As a next step, we plan to carry out the same evaluations using mature myocardiocytes.

There is no report on the development of myocardial mitochondria-targeting LP encapsulating RES in which the therapeutic effect on mitochondrial respiratory function was evaluated by a Seahorse XF analyzer. As the next step, we plan to study the efficacy of the β-MEND (RES) on some additional pathological models. We expect that the β-MEND described in this study will contribute the development of new and innovative cardioprotective agents in the future.

## 4. Materials and Methods

### 4.1. Reagents

RES was obtained from FUJIFILM Wako Pure Chemicals Co., Ltd. (Osaka, Japan). Egg yolk phosphatidylcholine (EPC) was purchased from the NOF Corporation (Tokyo, Japan). Sphingomyelin (SM), DC-Chol, N-(7-nitrobenz-2-oxa-1,3-diazol-4-YL)-dioleoyl-phosphatidyl-ethanolamine (NBD-DOPE) were purchased from Avanti Polar Lipid, Inc. (Alabaster, AL, USA). 1,1′-dioctadecyl-3,3,3′,3′-tetramethylindocarbocyanine perchlorate (DiI) was purchased from Takara Bio Inc. (Shiga, Japan). MTDR was purchased from Thermo Fisher Scientific Life Sciences (Waltham, MA, USA). All other chemicals that were used were commercially available and reagent grade products.

### 4.2. Preparation of LPs

LPs were prepared by the lipid film hydration method as previously reported [12]. In a glass tube, a total of 137.5 nmol of lipid was dissolved in ethanol, at a ratio of 3:4:3 of DC-Chol, EPC and SM as the molar ratio for the β-MEND. After adding 125 μL of chloroform and ethanol containing 25 nmol RES to the lipid suspension, a thin lipid film was formed by the evaporation of organic solvents for more than 2 h. Then, 250 μL of 10 mM 2-[4-(hydroxyethyl)-1-piperazinyl] ethane sulfonic acid (HEPES) buffer with pH 7.4 was added to the film in the glass tube, followed by incubation at room temperature more than for 15 min. After sonication of the tube for 60 s in a bath-type sonicator, the suspension was again incubated at room temperature more than for 15 min. The properties of the resulting LPs were measured by a Zetasizer Nano ZS (Malvern Panalytical, Worcestershire, UK).

### 4.3. Cell Lines and Culturing

H9c2 cells, rat cardiac myoblast cells, were obtained from the American Type Culture Collection (Manassas, VA, USA). These cells were cultured in Dulbecco’s Modified Eagle’s Medium High glucose (DMEM) (Sigma–Aldrich Co. LLC, St. Louis, MO, USA), which contained 10% fetal bovine serum (FBS) (Thermo Fisher Scientific Inc., Waltham, MA, USA) with penicillin and streptomycin.

### 4.4. FACS Analyses of LP Uptake

When preparing the LPs, 0.5 mol% of total lipids of DiI was added before the evaporation of the organic suspensions. About 1.0 × 10^6^ of H9c2 cells per well were seeded on a 6-well cell culture plate (Corning Inc., Corning, NY, USA) with FBS-containing DMEM and incubated overnight at 37 °C in an atmosphere containing 5% CO_2_. On the next day, following washing with phosphate-buffered saline without calcium chloride (PBS (-)), the media were replaced with the LP-containing DMEM without serum, with the final lipid concentration of 27.5 μM and incubated for 1 h. The cells were washed with PBS (-) and with PBS (-) including heparin (20 U/mL) twice, and then collected with trypsin 0.25% EDTA. After centrifugation at 300× *g*, 4 °C, for 5 min and removal of the supernatant, the collected cells were suspended in FACS buffer, which contained 5 mg/mL bovine serum albumin and 1 mg/mL sodium azide in PBS (-). Following filtering through a nylon mesh, the suspended cells were analyzed by CytoFLEX Flow Cytometer (Backman Coulter Inc., Brea, CA, USA). DiI was excited by 488 nm light, and the band pass filter for the fluorescence detection was set to 542–585 nm. The values for the intracellular uptake of each LP were expressed as the MFI, the integral values of the fluorescent intensity and the cell counts.

### 4.5. CLSM Observation of LP Uptake

When preparing the LPs, 0.5 mol% of total lipids of NBD-DOPE was added before the evaporation of the organic suspensions. A total of 2.0 to 5.0 × 10^5^ of H9c2 cells were seeded on IWAKI glass base dishes (AGC TECHNO GLASS Co, Ltd., Shizuoka, Japan) with serum-containing DMEM and incubated overnight under a 5% CO_2_ atmosphere at 37 °C. On the next day, after washing with PBS (-), the cells were incubated with the LP-containing FBS-free media for 1 h under 5% CO_2_ atmosphere at 37 °C, with a final lipid concentration of 55.0 μM. Following washing with PBS (-), the cells were incubated in serum-containing DMEM under 5% CO_2_ atmosphere at 37 °C. After a 2 h incubation, the cells were incubated for 15 to 20 min with 100 nM of MTDR in serum-containing DMEM. After washing the cells, the MTDR-containing media was replaced with a phenol red-free DMEM including FBS. The dishes were observed with a FLUOVIEW FV10i camera (OLYMPUS Corp., Tokyo, Japan), equipped with a water immersion objective lens with a magnification of 60-fold. The cells were excited with a 473 nm light for detection of NBD-DOPE and a 635 nm light for MTDR, and the band pass filters for these 2 fluorescent dyes were set to 490–540 nm and 660–760 nm, respectively.

### 4.6. Analyses of Mitochondrial Respiratory Function

A Seahorse XF HS Mini Analyzer (Agilent Technologies, Inc., Santa Clara, CA, USA) was used to assess mitochondrial respiratory function. In this experiment, 20,000 H9c2 cells were seeded on the Seahorse XF cell culture plate, which was then incubated overnight at 37 °C with 5% CO_2_. On the next day, after washing with PBS (-), the seeded cells were incubated with β-MEND (RES) with a lipid concentration of 55.0 μM in wells; naked RES with a concentration of 10 μM, or only DMEM without serum were used as non-treated controls. After a 3-hr incubation at 37 °C with 5% CO_2_, the media was replaced with the Seahorse XF assay media (Agilent Technologies, Inc.) to which glucose, pyruvate and glutamine were added and the plate was incubated again for 1 h at 37 °C without CO_2_. An assay was then performed using the Seahorse XF HS Mini Analyzer and a Seahorse XF cell mito stress test kit (Agilent Technologies, Inc.).

The cellular mitochondrial respiratory function was measured as OCR (pmol/min). The Seahorse XF cell mito stress test kit is composed from three reagents, oligomycin (an ATP synthase inhibitor of the electron transport system respiratory chain complex V), carbonyl cyanide 4-(trifluoromethoxy)phenylhydrazone (FCCP) (Uncoupler), and rotenone with antimycin A (inhibitor of electron transport system respiratory complex I/III), and these reagents were injected to each well to give the desired working concentrations (1.0 μM, 1.5 μM and 0.5 μM, respectively). Additionally, pyruvate was injected at the same time as FCCP in order to suppress the production of excessive spikes, in which the target concentration of pyruvate was 3.0 mM.

Following the assay, cells in each well were collected and counted by the CellDrop Automatic Cell Counters (DeNovix, Inc., Wilmington, DE, USA). The obtained OCRs were corrected by the cell number (pmol/min/10^4^ cells), as OCRs are known to be susceptible to the quantity of seeded cells in the same condition [30]. These cell-corrected OCRs were then corrected again by the values of the baseline OCRs of non-treated controls, and finally line graphs were obtained as the relative OCR values compared to the baselines of controls (% of Control Baseline). After plotting the measured OCRs on line graphs, “basal respiration”, defined as the difference in the OCRs between baseline and after treatment of rotenone with antimycin A, “maximal respiration” is the difference between and after injection of FCCP (the peak of the graphs) and after rotenone with antimycin A, and “spare respiratory capacity” is the difference between and after injection of FCCP and the baseline value. These parameters were expressed as bar graphs of the relative values when the non-treated control was expressed as 100% (% of control).

### 4.7. Evaluation of Cytotoxicity of LPs

The cytotoxicity of the β-MEND was assessed with the Premix WST-1 Cell Proliferation Assay System (Takara Bio Inc., Shiga, Japan). On the day before the assay, 30,000 H9c2 cells suspended in 100 μL of DMEM were seeded on a 96-well cell culture microplate (Greiner Bio-One International GmbH, Kremsmünster, Upper Austria, Austria) and incubated overnight at 37 °C in an atmosphere of 5% CO_2_. On the day of the assay, the seeded cells were transfected with naked RES, β-MEND (RES) and empty β-MEND, in which the final concentrations of RES were 5, 10, 20 and 30 μM in the wells. These reagents were added to 100 μL of DMEM without FBS first, and then 10 mM HEPES buffer at pH 7.4 was added to accommodate the volumes of the suspensions to a total of 150 μL. After a 3 h incubation at 37 °C with 5% CO_2_, the reagent-containing DMEM were replaced with FBS including Premix WST-1 reagent. After an additional 2 h incubation at 37 °C under an atmosphere of 5% CO_2_, the absorbance at 440 nm and 650 nm was measured by Varioskan LUX multimode microplate reader (Thermo Fisher Scientific, Waltham, MA, USA), respectively. The differences in the absorbance between at 440 nm and 650 nm mean cellular viability, and the values were expressed as the relative values when non-treated control was 100% (% of control).

### 4.8. Statistical Analyses

All data were expressed as the mean ± standard deviation (SD). For the comparison of the parameters of mitochondrial respiratory capacity by Seahorse XF Analyzer assays, the MFIs by FACS analyses and the relative absorbance as cellular toxicity assay, a non-repeated measures analysis of variation (ANOVA) was applied and followed by the Student–Newman–Keuls (SNK) test. When the two-tail *p* values were <0.05, the statistical analyses were determined as “significantly different”.

## Figures and Tables

**Table 1:** Properties of the β-MENDs.

Liposomes	Diameters (nm)	Polydispersity Index (PDI)	ζ-Potential (mV)
β-MEND (RES)	79.7 ± 10.2	0.26 ± 0.03	37.7 ± 8.6
Empty β-MEND	78.8 ± 10.0	0.26 ± 0.02	28.4 ± 11.1

Data are shown as the mean ± SD (n = 7–10).
